# Gouty arthritis: Can we avoid unnecessary dual-energy CT examinations using prior radiographs?

**DOI:** 10.1371/journal.pone.0200473

**Published:** 2018-07-10

**Authors:** Sivert Kupfer, Sebastian Winklhofer, Anton S. Becker, Oliver Distler, Christine B. Chung, Hatem Alkadhi, Tim Finkenstaedt

**Affiliations:** 1 Institute of Diagnostic and Interventional Radiology, University Hospital Zurich, University of Zurich, Zurich, Switzerland; 2 Department of Neuroradiology, University Hospital Zurich, University of Zurich, Zurich, Switzerland; 3 Department of Rheumatology, University Hospital Zurich, University of Zurich, Zurich, Switzerland; 4 Department of Radiology, University of California, San Diego, School of Medicine, United States of America; Charité, GERMANY

## Abstract

**Objective:**

The dual-energy CT (DECT) algorithm for urate detection is feasible only if hyperdense deposits are present. Based on our experience, around half of the performed DECT examinations show no such deposits and thus were useless for this indication. Our diagnostic accuracy study investigates whether conventional radiographs can serve as gatekeeper test prior to DECT for reliable exclusion of such radiopaque deposits.

**Materials and methods:**

In this retrospective study, 77 clinically indicated DECT examinations of the hand (n = 29), foot (n = 36) and ankle (n = 12) of 55 patients (13 female, mean age 62±15 years) with suspected gouty arthritis were included. Two blinded readers independently evaluated DECT, gray-scale CT images (reference standard) and corresponding standardized radiographs for the presence/location of dense soft tissue deposits.

**Results:**

Interreader agreement for detection of soft tissue deposits with DECT and radiographs was excellent (DECT: both readers, κ = 1; radiographs: both readers, κ = 0.94). DECT showed soft tissue deposits in 54/77 DECT (70%) scans. 30/54 scans (56%) showed deposits on the corresponding radiographs, while in 24 scans (44%) no deposits were seen on radiographs. Test performance of radiographs for soft tissue deposit detection: sensitivity 56%, specificity 100%, PPV 100%, NPV 48.9%, and accuracy 69%. Low density of the deposits was the main reasons for false-negative radiographs (19 cases, 79%), followed by superimposition of deposits by osseous structures (5 cases, 21%).

**Conclusion:**

Conventional radiographs of the hand, foot and ankle cannot serve as a gatekeeper test for reliable exclusion of radiopaque soft tissue deposits prior to DECT.

## Introduction

Detection of monosodium urate monohydrate (MSU) crystals is essential for the diagnosis of gouty arthritis [1]. In clinical practice, the reference standard for detection of urate crystals consists of polarization light microscopy after arthrocentesis [2]. However, this approach is invasive [3], there is a considerable likelihood of a "dry tap" during diagnostic arthrocentesis [4], and polarization light microscopy has a limited sensitivity of about 70% [5, 6]. The precipitated urate crystal deposits in advanced stages of gout disease can be detected on radiographs or conventional computed tomography (CT) images as radiopaque/hyperdense soft tissue deposits [7–9]. However, the imaging appearance of such dense deposits is nonspecific and they can be found in other crystal arthropathies like calcium pyrophosphate dihydrate deposition disease (CPPD), hydroxyapatite crystal deposition disease (HADD) [10, 11] and even as secondary crystal deposition in osteoarthritis [12]. Despite certain differences in average densities of these deposits [13], a pure assessment of density or morphology does not allow for a reliable distinction between urate- and non-urate containing deposits due to the large overlap of densities [14].

In contrast to conventional single-energy CT, dual-energy CT (DECT) uses two different x-ray spectra (high- and low voltage) and thus enables a non-invasive distinction of soft tissue deposits into those containing urate and those without [15–17]. The literature has shown that DECT features a considerably high sensitivity and specificity for urate detection of 71–100% and 85–100%, respectively, [1, 14, 18–21] if well-known artifacts are considered [22] and if it is taken into account that sensitivity of DECT decreases in patients with recent-onset gout [1, 23, 24]. However, it is only reasonable to perform a DECT in the work-up of gouty arthritis if hyperdense deposits are present. A recent study investigating the value of DECT for the work-up of gouty arthritis showed that in around half of the performed DECT examinations no such deposits were found and therefore the DECT examinations were useless [25]. To prevent unnecessary DECT examinations, a gatekeeper test is desirable for reliable exclusion of soft tissue deposits, which would save costs and reduce the radiation burden to the patients [2, 18, 26]. The STARD 2015 guidelines were applied for reporting of this diagnostic accuracy study [27].

In this study, we aimed to investigate whether conventional radiographs can serve as gatekeeper test prior to DECT for reliable exclusion of radiopaque deposits.

## Materials and methods

### Patient population

This retrospective study was approved by the Ethics Committee Zurich, Switzerland (BASEC-Nr. 2016–01994, December 22nd, 2016) and institutional review board. All procedures performed in this study involving human participants were in accordance with the ethical standards of the institutional and/or national research committee and with the 1964 Helsinki declaration and its later amendments or comparable ethical standards. Written informed patient consent requirement was waived by the Ethics Committee Zurich, Switzerland. Patients were not included when a general research rejection statement was present in their medical records.

**Fig 1:**
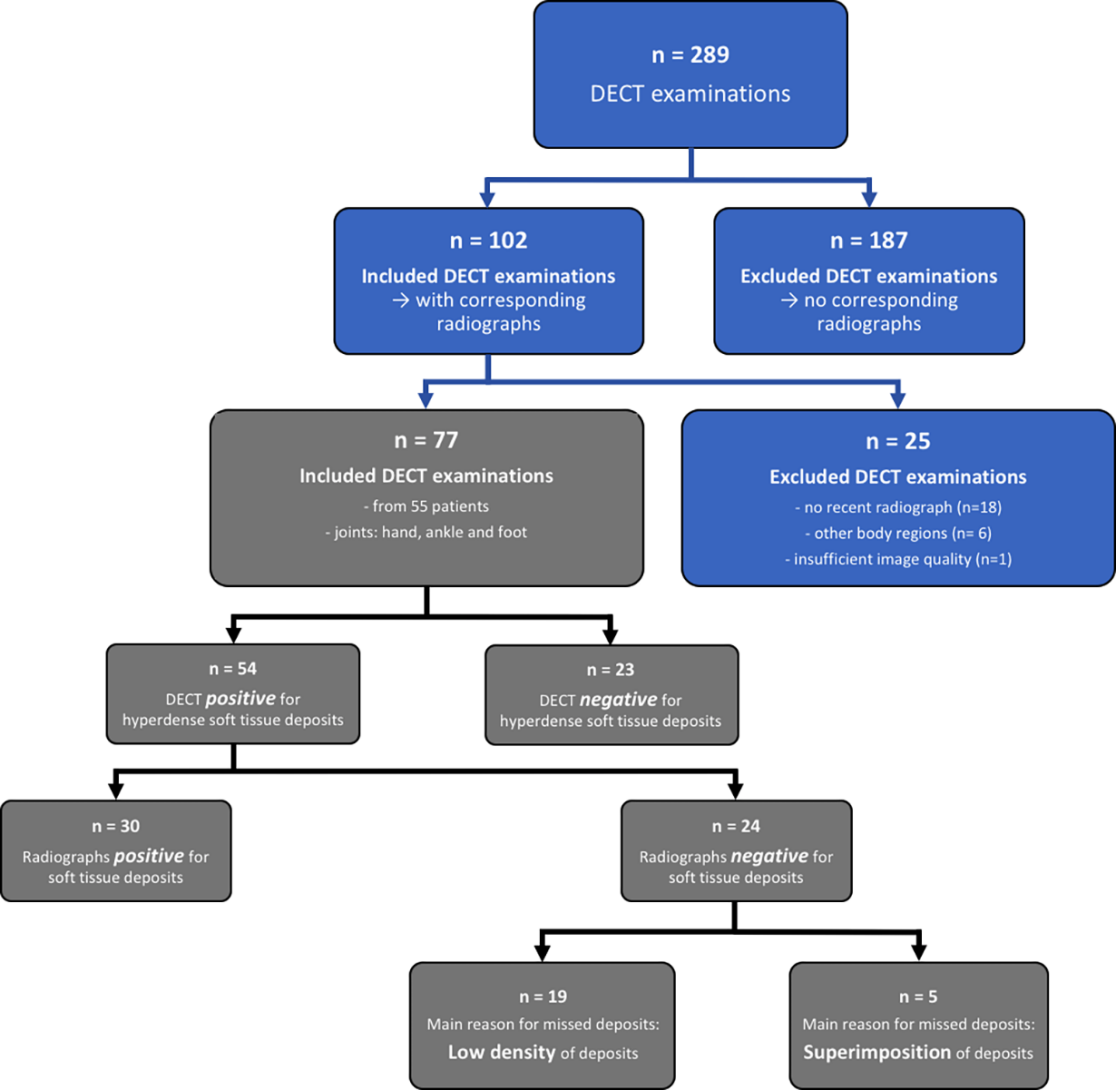
Flow chart of the study.

We screened 289 consecutive DECT scans of 211 patients (143 men, 68 women, mean age 60.4±15.7 years) with suspected gout that were performed between June 2013 and January 2017 in our radiology department (Fig 1). DECT was performed of the neck (n = 2), arm (n = 8), elbow (n = 5), hand (n = 101), hip (n = 2), knee (n = 14), ankle (n = 44) and foot (n = 113). All scans were clinically indicated. In all patients, DECT was performed when the standard diagnostic approach (arthrocentesis followed by polarization microscopy) of the referring clinicians was not feasible or inconclusive. DECT of the neck, arm, elbow, hip and knee were excluded because of the small number precluding meaningful statistics. Further exclusion criteria were non-available corresponding radiographs (n = 162), interval between DECT and corresponding radiograph > 3 months (n = 18), and insufficient CT image quality due to severe motion (n = 1). The radiographs were performed initially as part of the regular workup of patients with suspected arthritis at our institution.

Finally, 55 patients (13 females, 42 males, mean age 62.2±15.5 years) with suspected, not yet diagnosed (n = 34, 62%) and by their medical history known gout (n = 21, 38%) with a total of 77 DECT scans of the hand (n = 29), foot (n = 36) and ankle (n = 12) were included. *Known gout* was defined as gout being known from the patients' previous medical history anywhere in the body, which was based at that time on clinical symptoms, blood analysis, and/or arthrocentesis.

### CT data acquisition and image reconstruction

The CT scans serving as the reference test were performed using second- and third generation dual-source CT (SOMATOM Definition Flash/Force, Siemens Healthcare, Forchheim, Germany). Tube A and B potentials were 80/100kVp and 140/150kVp, the latter with tin filtration. Depending on the scanned body region, quality reference tube current time products of DECT ranged between 130-170mAs for tube A and between 200-250mAs for tube B. Automated attenuation-based tube current modulation was used in all scans. The average radiation doses of DECT, taken from the electronically logged patient protocols, were as follows: Average volume CT dose index (CTDI_vol_) hand: 6.7 mGy and ankle/foot: 10.3 mGy.

Axial images with a soft tissue convolution kernel (I50s, sinogram-affirmed or advanced modeled iterative reconstruction, strength level 3) were reconstructed with single and weighted energies (Dual-energy composition factor 0.5) with a slice thickness of 0.75mm (increment 0.5mm). Based on the weighted DECT images (50% 80kVp and 50% 140kVpSn) using a bone tissue convolution kernel (Q30s), regular morphologic gray-scale reformations in all three orientations with a slice thickness of 1 mm (increment 1 mm) were reconstructed. Reconstruction parameters were used as follows: minimum (HU) 150, iodine ratio 1.4, air distance 5, bone distance 10, resolution 4 and a material definition ratio 1.25. Post-processing of DECT was performed using dedicated software (syngo.via VA31, syngo Dual Energy Gout, Siemens), which color-codes urate crystal deposits as green. For each joint DECT post-processing took approximately 2 minutes.

### Radiographs

Digital radiographs as the index test were performed on our routine x-ray machine (Fuji, FDR AcSelerate, USA) equipped with a CsI detector and using standardized techniques. Depending on the joint an energy of 50–56 kVp and phototiming to set the tube current and exposure time (range 1.5–3.6 mAs) were used. Projections included standard dorsopalmar/dorsoplantar projections for the hand and foot and anteroposterior as well as lateral projections for the ankle.

### CT data analysis

**Fig 2:**
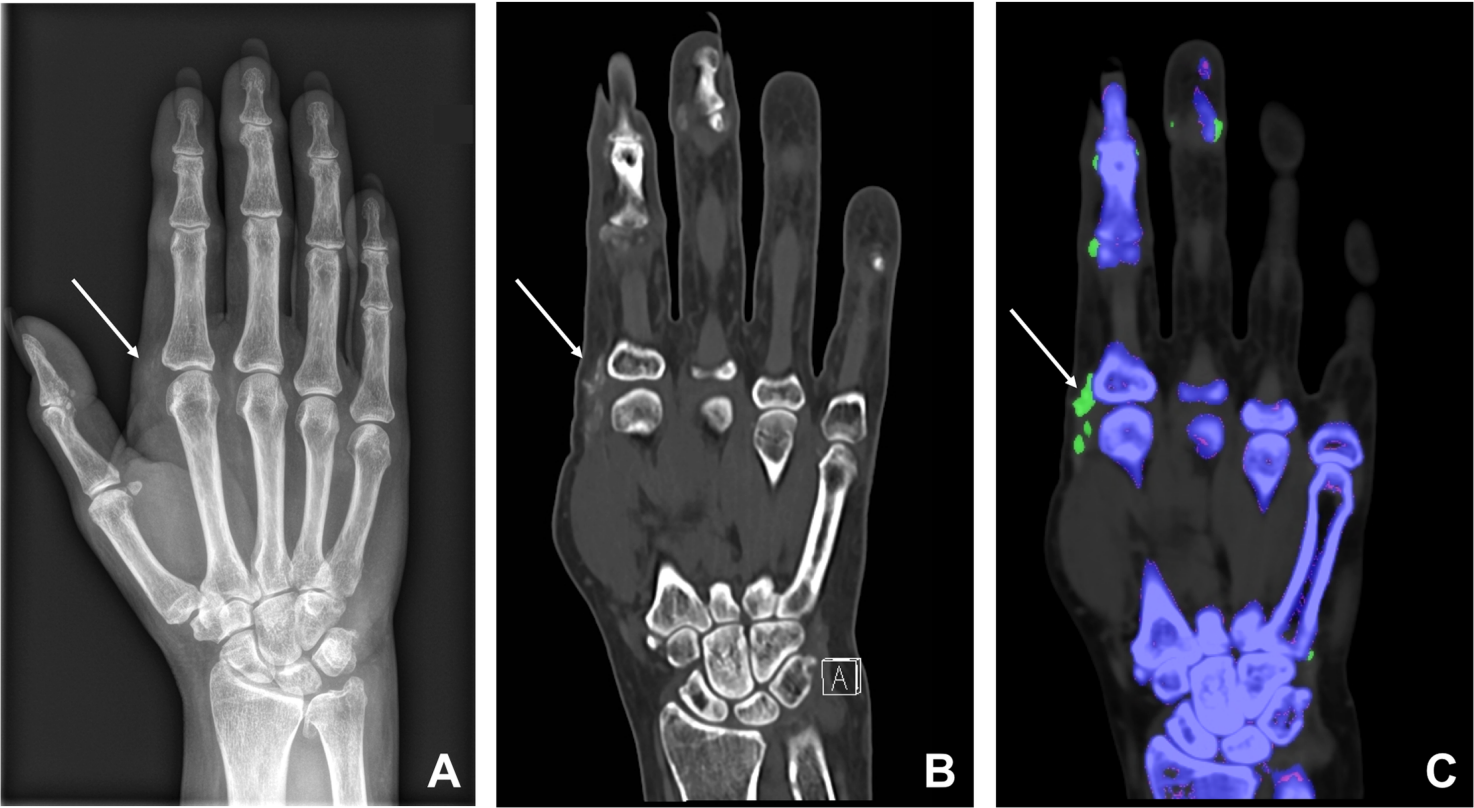
61-year-old woman with suspected gout. Radiograph (A) in dorsopalmar projection shows soft tissue deposits on the radial side of the metacarpophalangeal joint of Dig. II (arrow). Coronal reformations (B) of the corresponding gray-scale CT confirm these hyperdense soft tissue deposits (arrow). Coronal reformations of color-coded DECT images (C) indicate that the soft tissue deposits contain urate crystals.

Two readers (Reader 1 (R1) with 6 years (TF) and Reader 2 (R2) with 1 year (SK) of experience in radiology, respectively) blinded to the patients’ medical records independently evaluated the DECT images in random order as follows: location, presence or absence of soft tissue deposits (target condition) on gray-scale CT reformations and presence or absence of urate crystal deposits on color-coded images (multiplanar reformations and volume-rendered 3D images, Fig 2) were rated. The regular gray-scale CT images served as the reference standard for detection of hyperdense deposits. Both readers were fully aware of the known artifacts of DECT for urate deposit detection (e.g. false-positive findings of the nail bed and skin) [22] and of the potential underestimation of urate deposits by DECT [28, 29]. In addition, both blinded readers evaluated corresponding radiographs (index test) in standard projection/s for the presence and location of soft tissue deposits (target condition) 4 weeks after DECT readout to prevent recall bias.

Finally, in an unblinded fashion, both readers evaluated both datasets (DECT and radiographs) simultaneously to determine reasons why soft tissue deposits were seen on DECT images but not on radiographs, with the two following main categories: 1) superimposition of the soft tissue deposits by osseous structures or 2) particular characteristics of deposits (i.e. small difference between the density of the deposits and the summated density of the adjacent soft tissue penetrated by the x-ray beam and ill-definition of the deposits; both characteristics are summarized by the term “low density”) primarily hampered the visualization on radiographs. In cases of disagreement a consensus reading of both readers to revisit disparate reads was performed.

### CT attenuation measurements

After the readout, measurements of the attenuation (in HU) of the hyperdense soft tissue deposits were performed by using circular regions-of-interest (ROI). For the measurement, we used the mixed dual-energy CT images combining the two tubes voltages. Mean, maximum, minimum and standard deviation of attenuation in the ROIs were noted. Primarily, for the attenuation measurement the CT image of the deposit that was visible on the corresponding radiograph was used. For the deposits that were not visible on the corresponding radiographs the deposit with the most extensive burden was selected for attenuation measurements.

### Statistical analysis

Interreader agreement for qualitative measures including the detection of soft tissue deposits on radiographs, gray-scale CT images and for detection of urate crystal on color-coded DECT images was calculated using the Cohen's kappa (κ) coefficient: 0.21–0.40 indicated fair, 0.41–0.60 moderate, 0.61–0.80 good, and a kappa >0.81 indicated excellent agreement [30]. The difference in soft tissue deposit detection between radiographs and CT scans was calculated using chi-square test. We performed a subgroup analysis to determine whether there was a difference in deposit detection on radiographs between the DECT urate positive subgroup vs. those that were negative by using the Chi-square test.

We compared the deposit detection rate on radiographs between the different joints (hand, foot and ankle) by using the Fisher's exact test. To assess the performance of radiographs for soft tissue deposit detection, we compared the detection rate on radiographs with that from gray-scale CT, the latter severing as the reference standard. For performance testing sensitivity, specificity, positive prediction value (PPV), negative prediction value (NPV) and accuracy were calculated. For statistical analysis, commercially available software was used (IBM SPSS Statistics for Mac OS X, Version 24, IBM Corporation, Armonk, NY USA). Statistical significance was assumed at a two-tailed p-value below 0.05.

## Results

### Inter-reader analysis

The inter-reader agreement for detection of soft tissue deposits on radiographs, gray-scale CT images and for detection of urate crystal on color-coded DECT images was excellent (κ = 0.94, κ = 1 and κ = 1). In two cases, R1 and R2 had different assessments regarding the presence of deposits on radiographs. Consensus reading resulted in two positive cases showing deposits. Corresponding cases had hyperdense deposits on gray-scale CT images.

### Deposit detection performance

**Fig 3:**
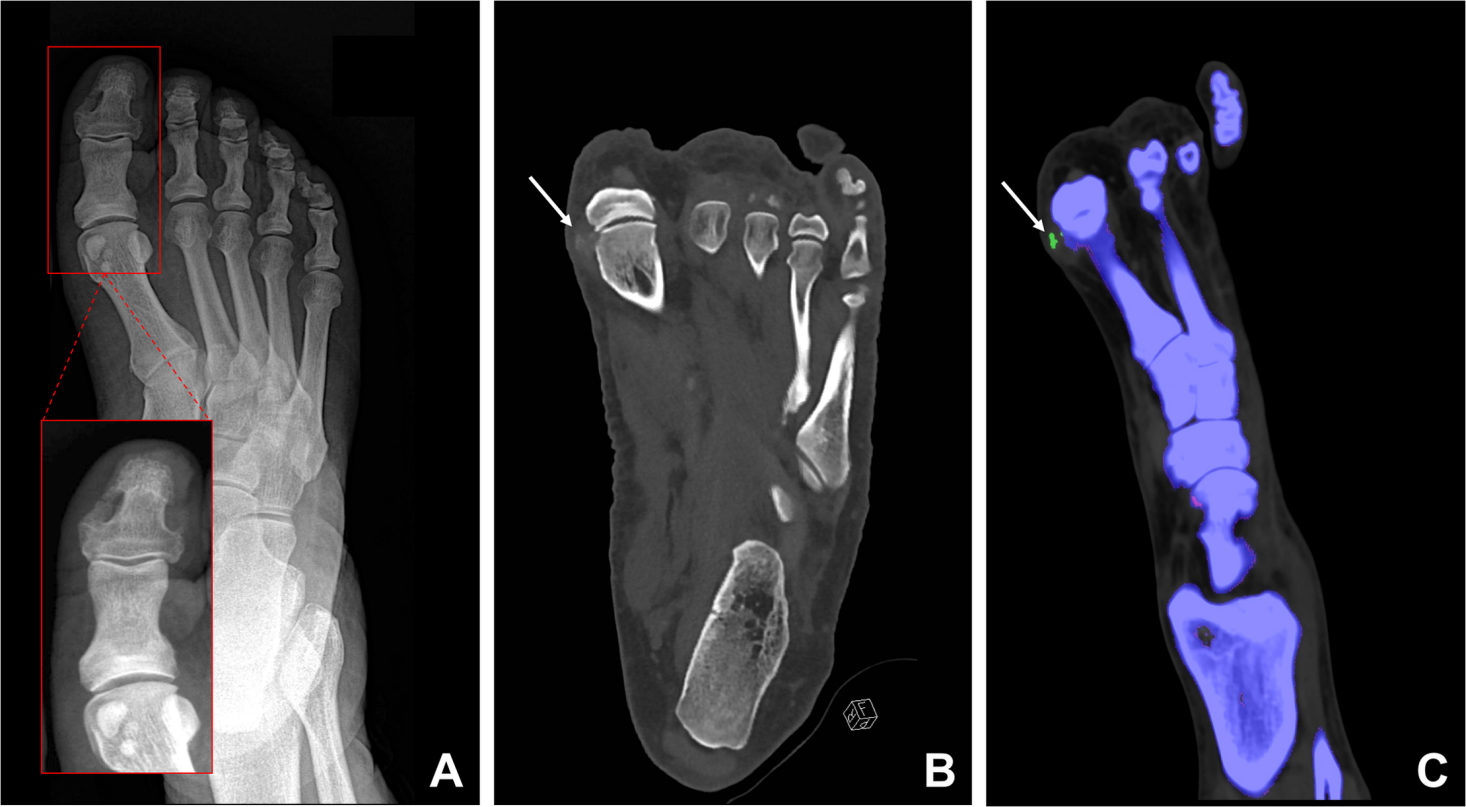
75-year-old man with known gout from his previous medical history. Dorsoplantar radiograph (A) shows no soft tissue deposits. Coronal gray-scale CT image depicts periarticular soft tissue deposits (B) medial to the metatarsophalangeal joint of Dig. I (arrow). Coronal color-coded DECT images (C) show that the soft tissue deposits contain urate crystals (arrow). The primary reason for the false-negative radiograph in this case was the low density of the deposit.

**Fig 4:**
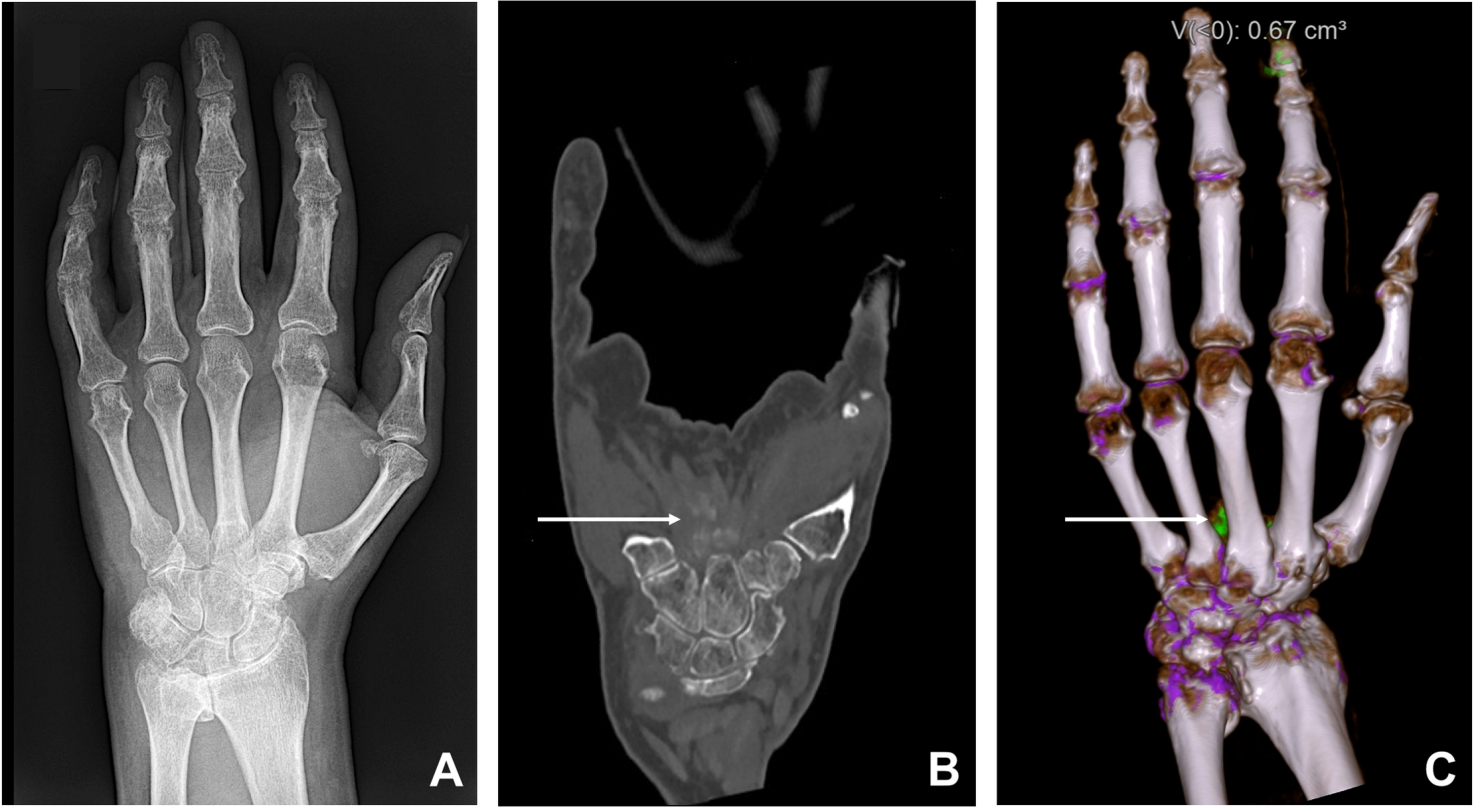
60-year-old man with suspected gout. Radiograph (A) shows no radiopaque soft tissue deposits. Coronal gray-scale CT images (B) demonstrate soft tissue deposits palmar to the carpus (arrow). Color-coded, volume rendered 3D DECT images (C) show that the soft tissue deposits contain urate crystals (arrow). The reason for false negative radiograph in this case was superimposition of the deposits by metacarpal bones.

In 54 of the 77 DECT scans (70%), hyperdense soft tissue deposits were found on regular gray-scale CT images (reference standard). On corresponding radiographs, these deposits were found in 30 cases only (56%, *true-positive*, Fig 2), whereas 24 (44%) radiographs showed no deposits (*false-negative*). The reason for false-negative radiographs was primarily the low density and ill-defined borders of the deposits (19 cases, 79%, Fig 3) and to a lesser degree the superimposition of deposits by osseous structures (5 cases, 21%, Fig 4). Twenty-three DECT scans and their corresponding radiographs were negative for deposits (*true-negative*). None of the radiographs showed a deposit that was not visible on DECT (*false-positive*).

### Diagnostic accuracy of radiographs

Based on these data the following performance characteristics of the index test radiographs for soft tissue deposit detection were calculated: sensitivity 56% (95% CI 42% to 68%), specificity 100% (95% CI 86% to 100%), PPV 100% (95% CI 89% to 100%), NPV 48.9% (95% CI 35% to 63%), and accuracy 69% (95% CI 58% to 78%).

#### Deposit detection performance: Subgroup analysis

In 30 of the 54 CT scans (56%) showing soft tissue deposits, dual-energy algorithm indicated urate crystals color-coded in green. No difference was seen in deposit detection on radiographs between urate-containing vs. non-urate containing CT (p = 0.787). There was no significant difference in deposit detection on radiographs comparing the different joints (hand, foot and ankle) (p = 0.34).

### CT attenuation measurements

No significant difference was seen between the density of the deposits that were visible on radiographs vs. those which were not visible on radiographs (189±58 HU vs. 195±75 HU, p = 0.74). Furthermore, there was no difference when including those deposits that were assigned to the group “low density” according to the radiographs (183±67 HU vs. 189±58 HU, p = 0.75). Additionally, there was no difference between the density of deposits that were considered as urate-negative vs. urate-positive (183±84 HU vs. 198±45 HU, p = 0.44).

## Discussion

Owing to inherent limitations, urate detection with DECT is feasible only when hyperdense soft tissue deposits are present that exceed a certain attenuation threshold [28, 31]. Thus, DECT performed for the detection and quantification of urate crystals in the absence of such hyperdense deposits represents an examination with limited or no diagnostic utility and significant costs (around 300$ for a peripheral joint [2]) as wells as unnecessary radiation exposure (albeit low, approximately 0.5 mSv) [18, 26]. Knowledge of presence of these deposits prior to DECT would be desirable. In our clinical experience, there is a considerable number of useless DECT examinations that lack these mandatory hyperdense soft tissue deposits. Therefore, we sought to investigate if radiographs used as a gatekeeper test prior to DECT could reliably exclude these unspecific deposits. If such radiopaque deposits would be accurately detectable on radiographs a consecutive DECT could justifiably follow. However, our results showed that radiographs had a sensitivity of only 56% for detecting those deposits whose presence was proven by gray-scale CT. This indicates that taking radiographs prior to DECT for this purpose is not appropriate.

According to our study the main reason for the high number of false-negative radiographs was the low density and ill-defined borders of the deposits (79%) and to a minor degree the superimposition of the deposits by osseous structures (21%). This is in line with previous studies showing that deposits in crystal arthropathies like gouty arthritis and CPPD may not be dense enough to be seen on radiographs [20, 32–35]. Along with the ill-defined borders of many deposits, the small difference between the density of the deposits and the summated density of the adjacent soft tissue is likely the reason for the hampered deposit detection on radiographs [36, 37]. The superimposition of the deposits by osseous structures caused false-negative radiographs in 21% which represents a well-known shortcoming of projection radiography. We found no difference regarding the detection rate on radiographs comparing the hand, foot and ankle. This is most likely, because the hand and foot have a comparable profile and diameters, and both were analysed on dorsoplantar/dorsopalmar projections only, according to the EULAR guidelines [38]. We might speculate that additional projections would have increased the deposit detection rate. However, on a lateral projection the x-ray beam passes a greater distance through tissue as compared to a dorsoplantar/dorsopalmar projection.

Beyond the primary goal of our study, we investigated whether there was a difference in the deposit detection rate between the urate-containing and non-urate-containing deposits. Previous literature indicated that the attenuation of MSU deposits on CT images is on average less dense (around 160 HU) compared to calcium-containing deposits in other crystal arthropathies such as CPPD (about 450 HU) [13]. Interestingly, our study showed no difference in the deposit detection rate on radiographs between the DECT urate positive and DECT urate negative subgroups. Correspondingly, we found similar attenuation values of the deposits in both groups. This might be explained by the complex changes of urate deposits that occur in the long term: some MSU deposits contain calcium initially [31] or may increasingly deposit calcium over time (especially in chronic stages), which in turn increases their density [39–41]. The mean density of urate containing deposits in our study was 198±45 HU which is higher than values reported in the literature (around 160 HU) [13].

We have to acknowledge the following limitations. First, the study population is relatively small and in our study 70% of the DECT scans featured hyperdense deposits compared to the mere 46% previously described. The most likely reason is that in our study only DECT scans with a preceding radiograph were included. Second, we included only some joints in our analyses (i.e., hand, foot and ankle), and results for other body regions and joints remain to be elucidated. Finally, we used standard radiography projections and techniques in our study only according to our institutional regulations, but additional, not routinely performed projections as well as new techniques as digital tomosynthesis could have improved the sensitivity of radiography for detecting soft tissue deposits.

In conclusion, our diagnostic accuracy study shows that in patients with suspicion of gout, radiographs of the hand, foot and ankle are not capable to reliably exclude radiopaque soft tissue deposits which are a premise for urate detection through DECT. Thus, radiographs cannot be used as a gatekeeper test for the decision if DECT should be subsequently performed.

## Abbreviations

CPPD: Calcium pyrophosphate dihydrate deposition disease
DECT: Dual-energy computed tomography
HADD: Hydroxyapatite crystal deposition disease
HU: Hounsfield units
MSU: Monosodium urate monohydrate
NPV: Negative prediction value
PPV: Positive prediction value
ROI: Region of interest
